# Effect of Cysteamine on Cell Growth and IgG_4_ Production in Recombinant Sp2.0 Cells

**Published:** 2015

**Authors:** Hoda Jahandar, Behrouz Vaziri, Leila Nematollahi, Tayebeh Afsharirad, Esmat Mirabzadeh, Fatemeh Torkashvand, Vahid Khalaj

**Affiliations:** Medical Biotechnology Department, Biotechnology Research Center, Pasteur Institute of Iran, Tehran, Iran.

**Keywords:** Cysteamine, IgG_4_, Monoclonal antibody, Protein disulfide isomerase, Sp2.0 cells, Thiol reducing agents

## Abstract

The manipulation of redox potential in secretory pathway by thiol reducing agents can be a strategy to improve the production levels of disulfide-bonded proteins including recombinant antibodies. Here we have studied the influence of cysteamine on viability and the production level of IgG_4_ in Sp2.0 cells. For this purpose, the recombinant Sp2.0 cells producing an anti CD33 IgG_4_, were subjected to different concentrations of cysteamine. At concentrations of 2, 4 and 5 mM cysteamine, the secreted levels of IgG_4_ did not change significantly. However, in concentration of 7 mM cysteamine, a significant decrease was observed in IgG_4_ levels which may indicate the cytotoxicity of this compound in higher concentrations. Our results show that the cysteamine treatment reduces the cell viability in a dose-dependent manner. Also it was observed that 2 mM cysteamine had no late effect on IgG_4 _production level and only at day 3, this concentration of cysteamine decreased the cell viability significantly. To test whether the addition of cysteamine can affect the expression level of protein disulfide isomerase, RT-PCR analysis was carried out. The results revealed that cysteamine does not affect the PDI transcription and expression level of IgG_4_ in this type of recombinant cells.

## Introduction

In recent years, monoclonal antibodies (mAbs) have become invaluable tools both in diagnosis and treatment of various diseases (1, 2). The high demand for mAbs has led to various attempts to obtain efficient expression systems (3, 4). Mammalian cells are commonly employed for the production of large and complex biotherapeutics such as antibodies (5). Several strategies can be used to improve the productivity of recombinant proteins in animal cells. Recently, a great attention has been drawn to transcription, translation and secretion processes (6). It has been shown that the specific production of mAbs is not well-correlated with transcription level, and the post-transcriptional events are the limiting steps (7). 

Correct folding and assembly of newly made antibody in Endoplasmic Reticulum (ER) has an important role in secretory performance. Only properly folded proteins are transported along secretory pathway into the culture medium (8). Therefore, targeting secretory pathway is an appropriate strategy to overcome mammalian cell bottlenecks. Previous reports demonstrated that aside from over-expression of foldases and chaprones such as Protein Disulfide Isomerase (PDI) and heavy chain binding protein (BiP) (9-11) the secretory performance can be improved by engineering redox environment in the ER (12). Addition of reducing agents to culture medium and change in redox conditions can influence the disulfide bond formation of proteins (12-13). The detailed mechanism underlying the effects of thiol reducing agents has not yet been clearly understood but it has been suggested that the disruption of normal redox potential gradient in the secretory pathway can strongly induce PDI and some other ER-resident chaperones and foldases (14). In previous studies, it was demonstrated that the addition of reducing agents to the culture medium of protein-producing cells resulted in either enhanced or decreased secretion level of disulfide-bonded proteins (12-13). Hence, the effect of thiol reducing agents on the productivity of recombinant proteins seems to be dependent on several factors including cell types, expression levels and target proteins (3, 15-16). However, influence of redox condition on the secretion of antibodies has not been properly studied. Protein disulfide isomerase is responsible for the formation and isomerization of disulfide bonds during protein folding and secretion (17). Several studies have carried out to find a correlation between the expression level of PDI and the recombinant protein production, but the results were inconclusive (7). Various factors like Δ^12^-prostaglandin, tunicamycin, heat shock and thiol reducing agents have been shown to induce PDI mRNA. Some of these effects seem to be a result of Unfolded Protein Response (UPR) (18). 

Therapeutic mAbs can be designed to have different constant regions. Selection of the proper subclass in drug development is based on the intended mechanism of action. IgG_4_ is the most suitable isotype for targeted drug delivery due to its reduced effector functions (19).

Sp2.0 is currently one of the standard mammalian host cells for the production of mAbs. 25% of the monoclonal antibodies marketed in the United States or European Union are produced in Sp2.0 (20).

In the present study, we investigated the effect of cysteamine on the secretion of IgG_4_ isotype from Sp2.0 cells. An anti CD33 IgG_4_ was used as a model protein. Furthermore, in order to assess whether cysteamine can affect mRNA transcription level of PDI, RT-PCR analysis was performed. 

## Experimental

*Materials*

pFUSE-CHIg-hG4 and pFUSE-CLIg-hk vectors, were purchased from Invivogen (CA, USA). Lipofectamine, blasticidin and zeocin were obtained from Invitrogen (CA, USA). Synthetic DNA fragments were provided by Gene Ray Biotech (Shanghai, China). Plasmids and RNA were extracted using QIAprep Spin Miniprep Kit and RNeasy Plus Mini Kit, respectively both from Qiagen (CA, USA). All restriction enzymes and complementary DNA (cDNA) synthesis with RevertAid™ First Strand cDNA Synthesis Kit were obtained from Fermentas (Maryland, USA). High Pure PCR Template Preparation Kit (Roche Diagnostics GmbH, Mannheim, Germany) was used for genomic DNA isolation. Western blot analysis was performed using horseradish peroxidase (HRP) conjugated chicken Anti-Human IgG (Gallus Immunotech, USA) and ECL Western blotting detection kit (GE Healthcare, Piscataway, NJ). Amicon filtering system was purchased from Millipore (MA, USA). Cysteamine was obtained from Sigma (MO, USA). The quantitative measurement of IgG_4_ in culture supernatants was carried out using Enzyme-linked Immunosorbent assay Kit for Immunoglobulin G_4_ (Uscn Life Science Inc. Houston, USA).

*Cell line and media*

The Sp2.0-Ag14 cells were obtained from American Type Culture Collection (ATCC- CRL-1581). The cell lines were maintained in RPMI-1640 supplemented with 10% fetal bovine serum (FBS) (both from GIBCO-Invitrogen, CA, USA). 

*Construction of expression cassettes*

The chemically synthesized variable region genes of heavy (VH), [GenBank: M83098.1] and light (VL), [GenBank: M83099.1] chains of anti-CD33 antibody produced by M195 were inserted into pFUSE-CHIg-hG_4_ and pFUSE-CLIg-hk vectors. pFUSE-CLIg-hk contains the gene of constant region of light chain and blasticidin resistance gene as a selectable marker. pFUSE-CHIg-hG_4_ encodes the heavy chain gene of an IgG_4_ isotype antibody and zeocin resistance gene.

pFUSE-CHIg-hG4-VH was constructed by inserting variable region of heavy chain into the pFUSE-CHIg-hG4 using *Nhe*I and *EcoR*I enzymes. Similarly, the variable region of light chain cloned into *BsiW*I*/Nco*I digested pFUSE-CLIg-hk vector, yielding pFUSE-CLIg-hk-VL. The correct gene sequence of variable region was confirmed by digestion and sequencing. Transfection quality DNA was prepared using QIAprep Spin Miniprep Kit. DNA was analyzed by agarose gel electrophoresis and quantitated by spectrophotometry (absorbance at 260 nm). Purified vectors were linearized with *Not*I. The resulting construct was used for transfection to generate stably transformed Sp2.0 cell lines. 

*Cell culture*

The Sp2.0-Ag14 cells (ATCC- CRL-1581) were cultured in RPMI-1640 containing 10% FBS. The medium for culture maintenance of recombinant Sp2.0 cell lines (rSp2.0) was the same but with the addition of 50 µg/mL zeocin and 0.25 µg/mL blasticidin. Subcultivation was routinely performed three times a week at ratios ranging between 1:3 and 1:6 according to the cell density. Cells were grown at 37 °C in an incubator with humidified atmosphere of 5% CO_2_ in air. 

*Generation of stable cell lines*

To determine zeocin/blasticidin sensitivity, cultures were seeded at a density of 2 x 10^5^ cells/mL in 12-wells plate. Different concentrations of zeocin (0, 200, 300, 400, 600, 800, 1000 µg/mL), blasticidin (0, 0.5, 0.75, 1, 2, 3, 4, 6, 8, 10 µg/mL) and blasticidin/zeocin (0, 0.25/50, 0.5/50, 0.75/50, 0.25/100, 0.5/100, 0.75/100, 0.25/200, 0.5/200, 0.75/200, 1/200,0.75/300, 1/300 µg/mL) were added to the culture medium. The culture media were replenished every 2-3 days and the minimum concentrations that killed the cells within 1-2 weeks, were selected. 

The Sp2.0 cells were transfected by pFUSE-CLIg-hk-VL using Lipofectamine. Selection was carried out by seeding 2 x 10^5 ^cells per well in 12-wells plate containing RPMI-1640 supplemented with 10% FBS and 0.75 µg/mL blasticidin. Resistant cells were subjected to the next transfection round by pFUSE-CHIg-hG4-VH. The double stable cells expressing antibody were selected in the presence of 50 µg/mL zeocin and 0.25 µg/mL blasticidin for 2 weeks as described above.

To confirm the integration of the constructs into the transfectants’genome, the genomic DNA from untransfected or resistant Sp2.0 cells was extracted using High Pure PCR Template Preparation Kit. PCR amplifications were carried out using the specific primers (Table 1). PCR cycles were as follow: 30 cycles of 94 ºC, 2 min, 58 ºC, 30 sec and 72 ºC, 1.5 min.

The expected PCR product sizes were 420, 425, 1500 and 800 bp for variable region of light chain (VL), variable region of heavy chain (VH), heavy chain (HC) and light chain (LC) , respectively. The PCR products were analyzed by agarose gel electrophoresis. 

**Table 1:** The sequence of primers used for amplification of PDI, β-actin, Variable region of heavy Chain (VH), Variable region of Light Chain (VL), Heavy Chain (HC), Light Chain (LC) genes.

Gene	Sequence of forward primer	Sequence of reverse primer
PDI	5'-ACAGCTGGCAGGGAAGCTGA-3'	5'-AGCCTCTGCTGCCAGCAAGA-3'
β-actin	5'-GCAAGAGAGGTATCCTGACC-3'	5'-CCCTCGTAGATGGGCACAGT-3'
Variable region of heavy Chain (VH)	5'-TTGAGAGTAGATGGTTTGAGCCTGAG-3'	5'-GTGCTAGCTGAGGAGACGGTG-3'
Variable region of Light Chain (VL)	5'-TTGAGAGTAGATGGTTTGAGCCTGAG-3'	5'-CCACCGTACGTTTGATTTCCAG-3'
Heavy Chain (HC)	5'-TTGAGAGTAGATGGTTTGAGCCTGAG-3'	5'-GCCAGCTAGGACTCATTTACCCG-3'
Light Chain (LC)	5'-TTGAGAGTAGATGGTTTGAGCCTGAG-3'	5'-CTAGCTCCCTCTAACACTCTCCCCTG-3'

*Evaluation of expression*

*RT-PCR*

Total RNA was isolated from rSp2.0 cell lines using RNeasy Plus Mini Kit and then used for complementary DNA (cDNA) synthesis with RevertAid™ First Strand cDNA Synthesis Kit, according to the manufacturer’s instructions. VL and VH gene were amplified from the cDNA using specific primers (Table 1). Total RNA from untransfected cells was analyzed as negative control.

*SDS-PAGE and Western blot*

For detection of IgG_4_ expression, SDS-PAGE and Western Blot analyses were performed.

Supernatants from rSp2.0 culture media were harvested and concentrated 2.5 fold, using Amicon filtering system. The concentrated supernatants were subjected to SDS-PAGE analysis. 

Western blot analysis was also performed under both reducing and non-reducing conditions, utilizing a HRP-conjugated chicken Anti-Human IgG. The separated proteins were then transferred to a nitrocellulose membrane at 18 V for 1 hour. Membrane was blocked with 2.5% nonfat dry milk in Tris buffer saline (TBS)-0.05% Tween 20 for 2 h. For detection, blots were incubated with the antibody at a dilution of 1:1500 in the blocking solution for 2 h at room temperature.

Chemiluminescent development of the HRP-conjugated antibody was achieved using the ECL western blotting detection kit. Concentrated culture supernatant of untransfected Sp2.0 cells was used as the negative control.

*Cultivation*

For cell culture, exponentially growing stable rSp2.0 cells at a density of 2× 10^5^ cells/mL were seeded in duplicate into 12-well plates. Cultures were maintained at 37 °C in humidified 5% CO_2_ incubator. When cell density reached approximately 4× 10^5^ cells/mL, the maintenance medium was replaced with fresh medium containing different concentrations (0, 2, 4, 5, 7 mM) of cysteamine. After 24 hours, cell count and viability was determined. Cells and culture supernatants were stored at -70 ^o^C for future analysis.

For late effect assessment, cells were seeded at 2 × 10^5 ^cells/mL in duplicate. Cysteamine was added to the culture medium at final concentrations of 2 mM. For 5 days, Culture plates were sampled daily to determine cell viability. Supernatants were aliquoted and kept at -70 ^o^C for subsequent antibody assays at the end of the experiment. Untreated rSp2.0 was used as control. Replicate experiments were performed independently. 

*Analytical methods*

Viable cell number was estimated using the trypan blue dye exclusion method. Secreted antibody concentration was measured by an Enzyme-linked Immunosorbent assay Kit for Immunoglobulin G_4_ according to the protocol provided by the manufacturer. Each sample was assessed in duplicate. Graghpad prism software (version5, La Jolla, CA, USA) was used for statistical analysis. Data were compared by two-tailed unpaired t-test. 

*Analysis of PDI mRNA*

To determine the effect of cysteamine on the transcription level of PDI, possible changes in the PDI mRNA were analyzed by RT-PCR. Total RNA was isolated from cysteamine treated and untreated rSp2.0 using an RNeasy Plus Mini Kit. First-strand cDNA was synthesized from 1 µg of total RNA using the RevertAid™ First Strand cDNA Synthesis Kit. β-actin mRNA was used as an internal control for PDI mRNA analysis (Table 1). PCRs were performed as 30 cycles of 95 ^o^C for 1 min, 58 ^o^C for 30 s and 72 ^o^C for 30 s. The predicted size of PCR products for PDI and β-actin were 186 bp and 318 bp, respectively. RT-PCR analysis was performed three times with RNA samples prepared independently. Quantity One®software (version 4.6.3, Bio-Rad Laboratories, USA) was used for semiquantative analysis of RT-PCR Products. The density (intensity/mm^2^) of PDI band was measured by the software and then normalized against actin band.

## Results

*Gene cloning and vectors construction*

Variable regions of light and heavy chain were successfully cloned in pFUSE-CLIg-hk and pFUSE-CHIg-hG4 vectors, respectively.

**Figure 1:**
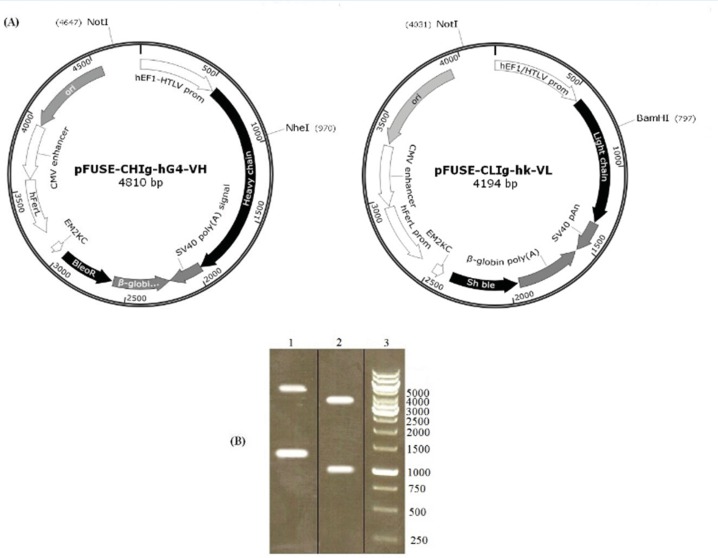
Analysis of recombinant vectors by restriction digestion. pFUSE-CHIg-hG4-VH and pFUSE-CLIg-hk-VL were cut by NheI/NotI and NotI/BamHI respectively. According to the vector maps (A), predicted product sizes were 1133 and 3677 bp for pFUSE-CHIg-hG4-VH and 960 and 3234 bp for pFUSE-CLIg-hk-VL. Digest reactions were electrophoresed on a 1% agarose gel (B). Lane 1, cut pFUSE-CHIg-hG4-VH; lane 2, cut pFUSE-CLIg-hk-VL; lane 3, 1kb DNA markers.

The restriction analysis of pFUSE-CLIg-hk-VL using *Not*I and *BamH*I resulted in expected band of 960 and 3234 bp. Similarly, the digestion of pFUSE-CHIg-hG4-VH with *Nhe*I and *Not*I showed expected bands at 1133 and 3677 bp (Figure 1). The constructs were finally confirmed by sequence analysis. 

*Establishment of producer lines*

**Figure 2:**
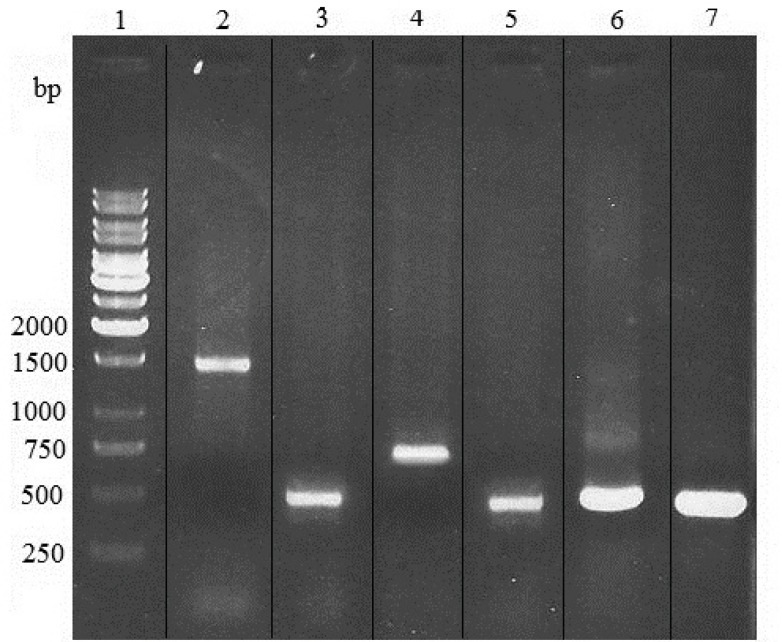
PCR analysis of genomic DNA from zeocin/blasticidin-resistant Sp2.0 cells. PCR products were electrophoresed on a 1% agarose gel. Lane 1, 1kb DNA markers; lane 2, amplification of heavy chain gene from DNA of resistant Sp2.0 cells; lane 3, amplification of variable region of heavy chain gene from DNA of resistant Sp2.0 cells; lane 4, amplification of light chain gene from DNA of resistant Sp2.0 cells; lane 5, amplification of variable region of light chain gene from DNA of resistant Sp2.0 cells; lane 6, amplification of variable region of heavy chain from recombinant vector pFUSE-CHIg-hG4-VH; lane 7, amplification of variable region of light chain from recombinant vector pFUSE-CLIg-hk-VL.

The optimal concentrations of blasticidin, zeocin and zeocin/ blasticidin for selection were found to be 0.75, 300 and 50/0.25 µg/mL, respectively. After transfection, in PCR analysis of zeocin/blasticidin-resistant cells, the amplified heavy chain, light chain, variable region of heavy chain and variable region of light chain products with predicted size of 1500, 800, 425 and 420 bp were observed (Figure 2). This confirmed the integration of pFUSE-CLIg-hk-VL and pFUSE-CHIg-hG4-VH into the genome of tested cells which were subsequently used in expression analysis. 

*Analysis of expression*

**Figure 3:**
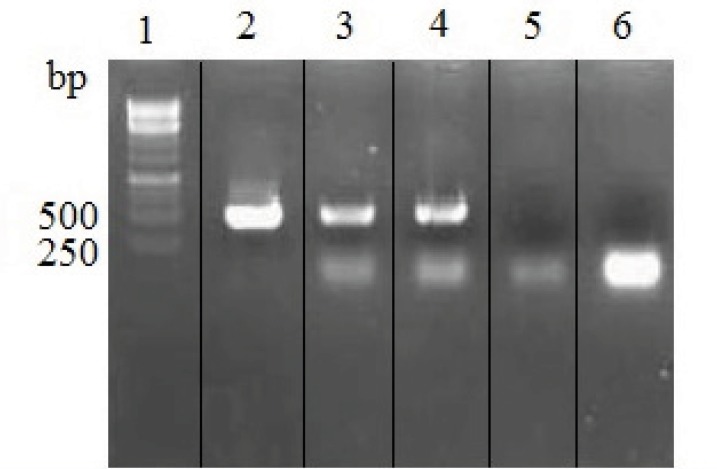
RT-PCR analysis of total RNA isolated from untransfected and rSp2.0. PCR products were electrophoresed on a 1% agarose gel. Lane 1, 1kb DNA markers; lane 2, amplification of variable region of heavy chain from recombinant vector pFUSE-CHIg-hG4-VH; lane 3, amplification of variable region of heavy chain gene from RNA of rSp2.0; lane 4, amplification of variable region of light chain gene from RNA of rSp2.0; lane 5, amplification of variable region of heavy chain gene from RNA of untransfected Sp2.0; lane 6, amplification of variable region of light chain gene from RNA of untransfected Sp2.0.

Transfected cells were analyzed for IgG_4_ expression at mRNA and protein levels. As it is shown in Figure 3, variable region of heavy and light chains were successfully amplified from rSp2.0 cells cDNAs. 

**Figure 4:**
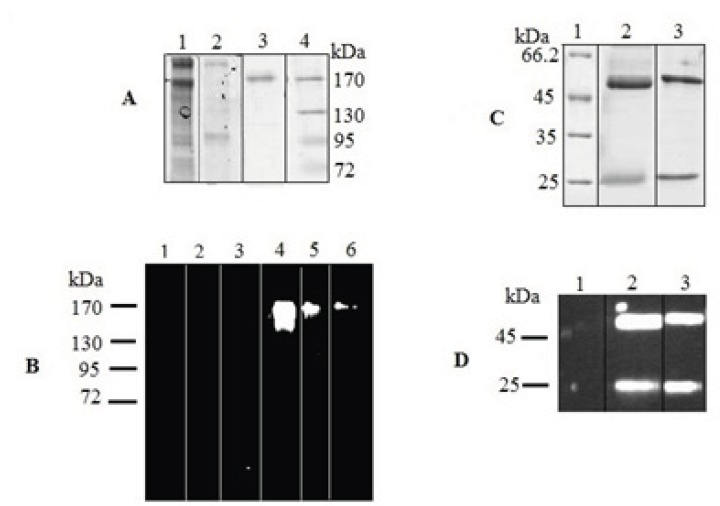
SDS–PAGE and Western blot analysis of expressed IgG_4_. The concentrated culture supernatant was separated on 8% resolving gel under non-reducing condition. Purified protein was analyzed under reducing condition on 12% resolving gel. The gels were stained with Coomasie Blue. In Western blot analysis, the blots were probed with a HRP-conjugated chicken Anti-Human IgG. Western blots were visualized by ECL. (A) Coomassie stained gel under non-reduced condition. Lane 1, supernatant of rSp2.0 cells; lane 2, supernatant of untransfected Sp2.0 cells; lane 3, positive control; lane 4, marker (B) Western blot analysis under non-reduced condition. Lane 1, marker; lane 2, supernatant of untransfected Sp2.0 cells (30 µg/mL); lane 3, supernatant of untransfected Sp2.0 cells (10 µg/mL); lane 4, supernatant of rSp2.0 cells (30 µg/mL); lane 5, supernatant of rSp2.0 cells (10 µg/mL); lane 6, positive control (C) Coomassie stained gel under reduced condition. Lane 1, marker; lane 2, purified IgG_4_, lane 3, positive control (D) Western blot analysis under reduced condition. Lane 1, marker; lane 2, purified IgG_4_, lane 3, positive control.

SDS-PAGE analysis revealed a protein with the expected molecular weight of 150 kDa in the culture medium of positive tranfectants (Figure 4A). Two expected bands of 25 and 50 kDa in reduced sample were also detected (Figure 4C). Further analysis by western blotting showed the reaction of a chicken anti-human IgG with these bands (Figure 4 B, D). These results indicated that chimeric IgG_4 _ has been successfully expressed in rSp2.0 cultures as a tetramer form.

*Effects of cysteamine on IgG4 production and cell growth*

*Cysteamine does not increase the levels of secreted IgG*_4_

**Figure 5:**
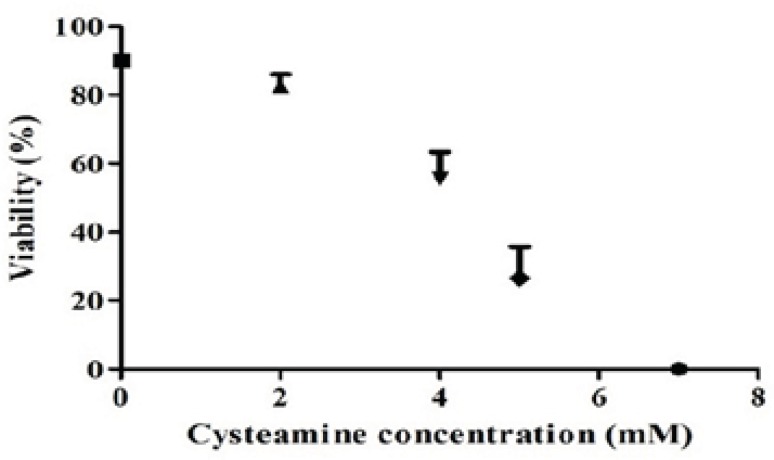
Effect of cysteamine on viability of rSp2.0. Stable rSp2.0 Cells at cell density of 4 ×10^5^ cells/mL were treated by various concentrations of cysteamine (0, 2, 4, 5 and 7 mM). The viability was measured after 24 h using the trypan blue dye exclusion method. Error bars represent the standard deviations calculated from the data obtained in three independent experiments.

**Figure 6:**
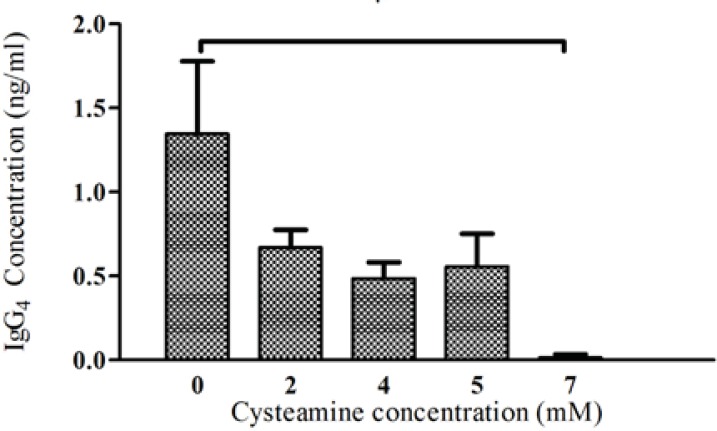
Effect of cysteamine on IgG_4_ production. Cysteamine in various concentrations (2, 4, 5, 7 mM) was added to medium of rSp2.0. The supernatants were collected after 24 hours and the amounts of secreted IgG_4_ were analyzed by ELISA. Error bars represent the standard deviations calculated from the data obtained in three independent experiments. *P<0.05, two-tailed unpaired t-test

To analyze the effects of cysteamine on the level of recombinant anti-CD33 IgG_4_ production, four different concentrations of this compound (2, 4, 5, 7 mM) were used. In concentration of 2 mM, cysteamine did not significantly influence the viability after 24 h. However, when the cysteamine was added in concentrations higher than 2 mM, the viability was reduced markedly. For instance, in concentrations of 4-7 mM, cell viability was decreased between 35-90% (Figure 5). The production levels in the presence of 2, 4, and 5 mM cysteamine were shown to be insignificantly different compared to the control. Addition of 7 mM cysteamine resulted in a significant decrease in the IgG_4_ levels from 1.346 ± 0.43 ng/mL to 0.014 ± 0.02 ng/ mL (Figure 6). 

**Figure 7:**
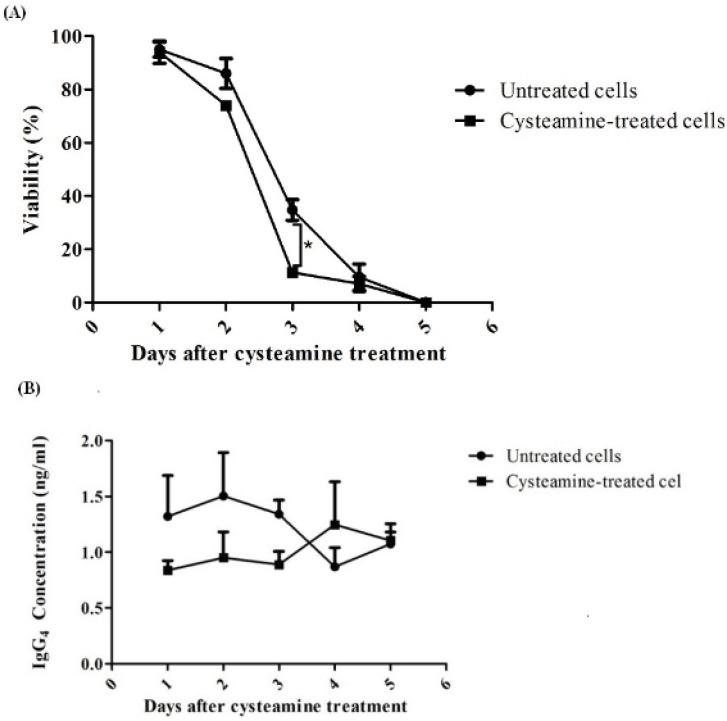
Assessment of late effect of cysteamine on rSp2.0. Cells were treated by 2 mM cysteamine. For 5 days cell viability (A) and IgG_4_ concentration (B) were determined daily. The amounts of secreted IgG_4_ were analyzed by ELISA. The viability was measured using the trypan blue dye exclusion method. *P<0.05, two-tailed unpaired t-test.

In another experiment, rSp2.0 was treated by 2 mM cysteamine which did not have any negative effect on viability in 24 h. The levels of secreted IgG_4_ and viability of treated and untreated (control) cells were monitored for 5 days. It was observed that antibody levels were insignificantly different between treated and untreated groups (Figure 7B). The same results were obtained for viability. However, at day 3 the viability of treated cells was decreased significantly (*p*=0.016) (Figure 7A).

*Cysteamin has no effect on the mRNA levels of PDI*

**Figure 8:**
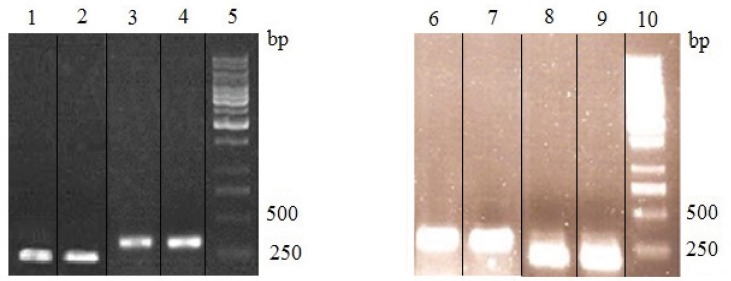
RT-PCR analysis of PDI mRNA in cysteamine treated rSp2.0. Lane 1, amplification of PDI gene from RNA of control cells; lane 2, amplification of PDI gene from RNA of treated cells by 2 mM cysteamine; lane 3, amplification of β-actin gene from RNA of control cells; lane 4, amplification of β-actin gene from RNA of treated cells by 2 mM cysteamine; lane 6, amplification of β-actin gene from RNA of control cells; lane 7, amplification of β-actin gene from RNA of treated cells by 4 mM cysteamine; lane 8, amplification of PDI gene from RNA of control cells; lane 9, amplification of PDI gene from RNA of treated cells by 4 mM cysteamine and lanes 5,10, 1kb DNA ladder. Cysteamine was added to culture medium. After 24 h, cells were harvested and total RNA was isolated from rSp2.0 cells. Levels of mRNA were standardized by β-actin mRNA. The expected size of products was 186 bp for PDI and 318 for β-actin. The experiments were repeated three times independently.

Previous studies have shown that in yeast, the PDI expression is immediately induced as a response to DTT treatment (14). To test whether the PDI expression was affected by cysteamine, RT-PCR analysis was performed as described above. The mRNA content of *β*-actin was used as an internal control. Figure 8 shows the mRNA content of treated cells with 2 and 4 mM cysteamine. It is expected that the mRNA level of PDI increases in the presence of cysteamine. However, semiquantitative analysis of RT-PCR products by comparing band densities of related fragments demonstrated no detectable differences in the amount of the PDI mRNA in the treated cells compared to the control (PDI/actin ratio was calculated ~0.97 for both treated and untreated cells) (Figure 8).

## Discussion

The production of recombinant proteins using various expression systems is a complex process and numerous approaches have been used to increase the yields of heterologous protein production (3, 21-22). 

It has been noticed that the expression of disulfide-bonded proteins such as IgG_4_ is limited by assembly and secretion steps (12). Thus engineering of these processes may improve the productivity. For this purpose, several studies have focused on two main approaches: overexpression of chaprones and foldases, mainly PDI (9-10) and engineering of redox potential of secretory pathway that suggested may indirectly induce PDI and other ER-resident proteins (14).

Previous studies have demonstrated that the addition of reducing agents resulted in either enhanced or decreased secretion levels. Alberini *et al*. (1990), reported that in the presence of 2-mercaptoethanol, the secretion of immunoglobulin M assembly intermediates was increased in B and plasma cells. This improvement is supposed to be a result of disruption in normal redox potential gradient of the secretory pathway. This can trigger the unfolded protein response and secretion of retained proteins (14). Also it was shown that the PDI1 gene of yeast, as an initial response, is strongly (>2.5-fold) induced by DTT (14).

The beneficial effects of reducing agents on the expression level of proteins may not be generalized. There are some reports which indicate that the reducing agents may decrease the secretion of disulfide-bonded proteins (13). To test the hypothesis that whether cysteamine could increase IgG_4_ secretion in recombinant Sp2.0, the present study was designed. The results showed no significant change in antibody secretion levels after 24 h of adding 2, 4 and 5mM cysteamine. Also 2 mM cysteamine did not have any late effect on IgG_4_ concentration in the culture medium. These results may be due to clonal variation of IgG_4_ and PDI expression levels in cell populations of rSp2.0 cells (16). It has been observed that the same experiments may lead to different results in high and low producing cell lines (7, 16). Subclass of antibody is another parameter that may influence the obtained results. Hayes *et al*. (2010) have found that the overall IgG_4_ productivity is not controlled by PDI in CHO and NSO cells (7). However, a number of experiments indicate that the upregulation of PDI has a positive effect on expression levels of other subclasses (9).

We observed that addition of 7 mM cysteamine resulted in decreased IgG_4_ concentration. This may be due to strong cytotoxic effect of cysteamine at this concentration.

Cysteamine showed toxic effects in a dose-dependent manner. Although exposure to 2 mM cysteamine did not affect the viability in 24 h but the viability was significantly decreased at day 3. This may be resulted from more sensitivity of rSp2.0 cells to cysteamine in stationary phase compared to the exponential phase (growth dependent sensitivity) (23).

It has been shown that the PDI gene can be strongly induced by DTT during the first hours of exposure (14). In this sense, we analyzed any possible changes in expression level of PDI in treated cells. As the results showed, cysteamine did not affect the transcription level of PDI at low and moderate concentrations. Our findings are in agreement with the results presented by Hayes *et al*. (2010). They demonstrated that the PDI overexpression or silencing had no effect on secretory IgG_4_ levels. 

In conclusions, we analyzed the effect of a reducing agent, cysteamine, on the production level of a chimeric anti-CD33 IgG_4_. The results showed that cysteamine does not increase IgG_4_ production levels in rSp2.0 but has a dose dependent negative effect on the cell viability. It was also demonstrated that PDI transcription is not induced in response to cysteamine addition. The lack of enhancing activity of this compound on the productivity may indicate the host to host and protein to protein variations.
